# Green innovation and enterprise digital transformation: Escape from the “dilemma” of development and governance choices

**DOI:** 10.1371/journal.pone.0301266

**Published:** 2024-05-16

**Authors:** Jinghuai She, Qi Zhang

**Affiliations:** College of Business Administration, Capital University of Economics and Business, Beijing, China; Shanghai Business School, CHINA

## Abstract

The digital economy is now the expected norm for economic development, warranting strategic importance for enterprise digital transformation. Nonetheless, enterprises have a lengthy journey to embark upon for digital transformation. On the one hand, resource-based demands pose a significant challenge due to the development characteristics of the initiative; on the other hand, excessive emphasis on economic gains may result in severe environmental issues. Therefore, this paper examines whether green innovation, which combines environmental and economic benefits, can effectively address the above dilemma. The study includes all A-share listed companies from 2010 to 2020 as the research sample, and empirically investigates the impact of green innovation on enterprise digital transformation and its mechanism based on resource-based view. The study concluded that (i) green innovation has a significant positive impact on corporate digital transformation performance, exhibiting asymmetric effects. The robustness tests confirmed the validity of the findings. (ii) Enterprises that actively engage in green innovation can effectively reduce their financial constraints, enhance their operational capacity, and enable the efficient allocation of resources, thereby promoting digital transformation within the enterprise. (iii) There is a regional imbalance in the conversion of green innovation performance into economic performance. The aforementioned results offer fresh insights for investigating the connection between green innovation and digital transformation. Additionally, these findings hold significant implications for the discourse on the synergistic advancement of the environment and economy.

## 1 Question raising

Enterprise development should be a comprehensive strategy. When the digital economy becomes a powerful force, serving as a crucial engine of economic development in the current post-epidemic era and the future [1, 2], enterprises will inevitably take on the task of digital transformation as the primary driver of economic progress. In January 2022, the State Council released the “14th Five Year Plan for the Development of Digital Economy” (Guo Fa [2021] No. 29, hereinafter referred to as the “Plan”), marking China’s first national special plan on digital economy. The “Plan” identifies the promotion of digital transformation and upgrading of enterprises as one of the eight key tasks. Digital transformation has become standard practice in the future development of enterprises.

The concept of digital transformation has been a contentious issue in academia since its inception at the start of this century. Scholars from a technological standpoint describe digital transformation as an organisation’s capacity to use the transformative and disruptive influence of a new age of digital technologies to enhance overall operational and business efficiency [3–5]. Scholars with a strategic development perspective, in addition to incorporating the theories of the technology-based perspective school, maintain that digital transformation is a process whereby organisations employ various digital technologies to dismantle “information silos”, consolidate internal and external resources, and elicit strategic responses aimed at achieving strategic change [6, 7]. With further research, scholars have now integrated the aforementioned perspectives and developed a more comprehensive definition of digital transformation. This definition emphasises that digital transformation is a complete management process in which enterprises utilise digital and computer technology to achieve disruptive changes in their original organisational elements, leading to organisational innovation, reconfiguration and development. This is likely the most widespread management challenge of the past and future [8]. Digital transformation is not solely a technology shift [9], but also holds considerable implications for numerous other facets of a business’s operations, potentially including all aspects [10].

As the development process of the global digital economy accelerates, enterprises must shift their focus from cost to efficiency. Therefore, digital transformation places pressure on enterprises to upgrade their resources and strengthen their innovation efforts [11]. 70% of digital transformations ultimately fail [12], Enterprise digital transformation is not only necessary for economic progress [13], but is also a driving force for production resumption and economic development in the post-epidemic era. However, it is inevitable that enterprises will face challenges during the difficult transition away from the “crude” development model. The crude approach of economic development is bound to incur a heavy cost of damaging the natural ecological environment. The lessons of centuries of experience demonstrate that the strategy of “prioritising development, over governance” is ineffective. Enterprises should not only promote digital transformation and cultivate their economic strength, but also participate actively in ecological and environmental construction, assume social responsibility, and seek a way to achieve long-term development in the future. Green innovation, which combines environmental and economic benefits [14], is widely regarded as a useful tool for enterprises to enhance production, increase efficiency and attain sustainable development. However, both green innovation and digital transformation exhibit high investment, high risk and low return traits, differing from the conventional shift of production mode. Based on the hypothesis of “bounded rationality of economic agents hypothesis”, decision-makers find it difficult to break out of the “environmental-economic governance cycle” and achieve coordinated governance of the environment and economy when facing the current situation of limited production factors in enterprises. The effectiveness of effectively mitigating the contradiction between environmental governance and economic development lies in the ability to appropriately handle the coexistence of green enterprise development and economic development. Currently, the “governing while developing” coexistence model is widely recognised by both academics and practitioners. This model provides some relief to the impasse between environmental governance and economic development, but it does not fundamentally solve the historical legacy of prioritising development over governance. Moreover, it is susceptible to weakening governance because fulfilling immediate needs for development can further widen the gap between environmental governance and economic development in the long term. In light of this, businesses aim to attain sustainable growth. The question then becomes: how to select a path towards development from its very foundation?

If the historical legacy issues of prioritising development over governance continue to persist, perhaps it would be more beneficial to consider the concept of “governing while developing.” In this way, we can eliminate pollution at the source and optimize production through green innovation to enhance sustainable development efficiency. This will promote the digital transformation of enterprises, further accelerating the pace of economic resumption during the post-epidemic era of work and production resumption. Carrying the expectations of scientific and technological innovations as well as green concepts, cultivating green innovations has always been a crucial approach towards alleviating the conflict between rapid economic growth and serious environmental pollution [14, 15]. Unlike traditional technological innovation, green innovation involves the development and implementation of commercially viable practices, procedures, systems, and products with the aim of achieving sustainable development. This approach not only yields more precise ecological benefits [16] but also generates substantial economic benefits [17, 18], therefore, it holds long-term significance. However, it has been argued by some that the long-term advantages of green innovation in economic growth have increased more slowly than its drawbacks [19, 20]. To enhance the understanding of the link between green development and economic growth, this study chooses all A-share listed firms between 2010 and 2020 as the research subject. Utilising the resource-based view, we employ a high-dimensional fixed-effect model to examine the influence of green innovation on the digitalisation of enterprises. Abbreviations of technical terms will be explained upon first use. It further investigates the mediating role of green innovation in enterprise digital transformation via an intermediate mechanism regression model.

The potential marginal contributions of this study are twofold. Firstly, it presents new research opportunities for investigating environmental governance and economic development in a mutually harmonious and peaceful manner. Building on the notion of “governing while developing”, the study validates the feasibility of this approach for enterprises. Secondly, it examines the “black box” mechanism of green innovation and its correlation with digital transformation within enterprises. The identification of the intermediary mechanisms in this study can reinforce the theoretical underpinning supporting the correlation between green innovation and enterprise digital transformation. In addition, it can offer practical guidance for enterprises seeking to achieve digital transformation and sustainable development.

## 2 Theoretical analysis and hypothesis proposal

### 2.1 Green innovation and enterprise digital transformation

Existing research has extensively investigated the driving factors behind digital transformation in businesses. Externally, institutional surroundings, such as state financial subsidies [21], tax incentives [22], open data access [23], financial support and its market-based reform [24, 25], alongside external factors like the market economic environment [26], market venture capital intervention [27], industry competition [28, 29], and supply chain finance [30], all have a crucial role in propelling digital transformation. Internally, digital transformation is facilitated by a range of factors including financial performance [31], executive team heterogeneity and behaviour [32, 33], internal asset allocation [34], management of digital change [35], synergy [36], institutional investor heterogeneity [37], and other internal elements. Throughout this study, regardless of government financial and taxation policies, institutional support, market conditions, or internal talent and compensation, assets and other internal and external resources have emerged as the primary catalyst for digital transformation in this unyielding stage.

The resource-based view, developed based on Wernerfelt’s work (1984) [38], posits that the fundamental factor that results in differences between enterprises is their ability to allocate available resources. Additionally, the theory highlights that the resources of any given enterprise are limited. As previously mentioned, digital transformation refers to an organisational activity that utilises advanced digital technology to facilitate the implementation of disruptive changes to the original organisational components, resulting in innovative upgrades to the organisational structure. Digital transformation is a comprehensive modification that occurs throughout all levels of an enterprise, from top to bottom [39]. Hence, enterprises engaging in digital transformation typically require a substantial investment of resources [11]. It is evident that underpinning this transformation is a key weakness in the resource base of the enterprise. Enterprise resource management commonly experiences two core difficulties: One is the enterprise’s inherent limited resources, and the other is the challenge of enterprise resource allocation. Overcoming the resource base barrier is the precursor to any successful enterprise digital transformation. Green innovation, an innovation activity designed to enhance environmental benefits and create profound changes in technologies, processes, or products [40, 41], considers both environmental and economic benefits and has unique advantages in creating sustainable value. On one hand, green innovation shares many features of regular innovation, including the ability to create new technologies, products and strategies that can help enterprises overcome resource limitations and inject fresh resources into their operations. However, green innovation also includes a specific focus on sustainable value creation, which sets it apart from traditional innovation and enhances its importance to enterprises seeking to operate sustainably [42]. On the contrary, green innovation activities strive to conserve energy and cut emissions [43], trim production costs [44], and streamline business processes, which can significantly boost and refine the efficiency of distributing enterprise resources, and therefore alleviate the challenges of performing everyday business operations. In the context of addressing the ‘environmental pollution-economic development’ issue to urge national sustainable development, green innovation is regarded as a crucial means to achieve industrial transformation and upgrading [45, 46] enhance financial performance [46], and promote high-quality economic development [45]. Enterprises utilise green innovation as an effective strategy for technological advancement, leading to reduced consumption of production resources [16], increased production capacity [47], and decreased production costs [44, 48]. Additionally, within the context of worldwide efforts towards enhancing environmental concerns, green innovation can optimise business processes and improve the efficiency of enterprise resource allocation, thereby supporting environmental protection. Meanwhile, amidst the global effort to improve environmental issues, enterprises can utilize green innovation to effectively control pollutant emissions using clean production methods. This practice, as shown by Du et al(2021) and Liu et al. (2020) [16, 47], sends positive signals to the government and the market while meeting the expectations of all stakeholders for green enterprise development. By doing so, businesses can garner the support and trust of all stakeholders and create a competitive edge in accessing external resources. Green innovation not only enables enterprises to generate and amass internal sustainable resources, but also facilitates external support, thus helping to overcome the constraints of limited resources and providing a robust backbone for the development of enterprise digital transformation. Furthermore, green innovation offers the distinctive dual advantage of acquiring external backing. In addition, the distinct dual features of green innovation allow it to integrate into the organization’s institutional system [49], boost organisational effectiveness, lessen personnel and institutional redundancy [50], optimize internal resource allocation effectiveness, enhance digital transformation efficiency, and align positively with the trend of the digital economy. In conclusion, it is considered that green innovation can effectively address the two predominant resource-based challenges encountered during enterprise digital transformation, aiding enterprises to overcome the resource-based dilemma and enabling further advancement of digital transformation. As a result, Hypothesis 1 is formulated.

Hypothesis 1: Under the same other conditions, the development of green innovation of enterprises will help accelerate the digital transformation of enterprises.

### 2.2 The mechanism of green innovation for enterprise digital transformation

Based on the resource-based view, combined with the results of the previous literature analysis, it is found that green innovation can improve the performance of digital transformation by helping enterprises break the “bottleneck” of their own resource limitations and improve the efficiency of transformation resource use. Incorporating green development into the economic transformation process is not solely a unique chance to make progress, but it is also an esteemed obligation entrusted by history [51–53]. On the one hand, through green innovation, enterprises create sustainable internal resources for digital transformation while creating favourable conditions for obtaining external resource support. On the other hand, through green innovation, enterprises optimise various business processes, make efficient use of existing resources, and lay a solid foundation for the active development of digital transformation. Therefore, the study will explore the intrinsic mechanism of green innovation affecting the digital transformation of enterprises from the two paths of resource base acquisition and resource base optimisation.

#### 2.2.1 Resource base acquisition mechanism

The resource base of an enterprise’s digital transformation usually comes from the enterprise’s internal resource stock and external resource support. According to the resource-based view, enterprises have limited resources of their own, and it is difficult to meet the needs of enterprises to carry out energy-consuming and high-risk organisational change activities. Therefore, when promoting digital transformation, enterprises should pay more attention to the process of acquiring and accumulating internal resources and external resources. A stable source of funding is a core component of an enterprise’s resource base, as well as an important prerequisite for ensuring the long-term development of its activities. Due to the existence of information asymmetry in the market and various types of agency problems [54], firms face different degrees of financing constraints. When enterprises face strong financing constraints [55], it is difficult to ensure a stable source of funds, which greatly limits their ability to cope with high costs and risks [56, 57], which will further weaken the incentives for enterprises to actively participate in digital transformation. In recent years, as society as a whole has paid more attention to the realisation of environmental and economic benefits for enterprises, the “green” signal emitted by enterprises has become an important factor in their ability to obtain internal and external funding [58]. Research by Chen and Shang (2023) [59] pointed out that one of the key criteria for the government to assess whether an enterprise has sustainable development values and whether it provides a series of reasonable guarantees, such as financial subsidies and tax incentives, to reduce financial distress is the amount it pays for the ‘green’ field. Lu and Li (2023) [50] also mentioned that government departments and financial institutions are more inclined to provide policy support to enterprises that carry out green innovation. In addition, from the perspective of market demand, green innovation can enhance product differentiation advantages by tailoring product development to consumer needs [53, 60]. This advantage is not only conducive to developing new markets and increasing market share, but also meets consumers’ pursuit and awareness of environmentally friendly products and brings more environmental premiums [61]. At the same time, “green investment” in the capital market also increasingly favours companies that are more likely to produce differentiated green products [62, 63]. To summarise the existing research, green innovation is an important embodiment of enterprises actively fulfilling their social responsibility, and to a certain extent, it can play the role of “endorsement”, which not only creates a good reputation and wins the favour of various stakeholders [64]; at the same time, due to the consumer’s choice of preference, it is helpful to urge enterprises to increase the supply of green products to improve internal operating capital income [58], thus reducing the threshold of enterprise financing and easing enterprise financing constraints, and ensuring strong capital reserves for enterprise digital transformation. In summary, Hypothesis 2 is proposed.

Hypothesis 2: If other conditions remain unchanged, enterprises actively carry out green innovation, which can effectively alleviate the constraints of enterprise financing, thus providing stable financial resource base support for enterprises’ digital transformation, and promoting enterprises’ digital transformation.

#### 2.2.2 Resource base optimization mechanism

Enterprises can obtain stable financial support is to lay the premise of its activities to carry out the resource base. At the same time, the ability to obtain and accumulate funds to be reasonably and effectively allocated is an important criterion to test whether the resource base ensures the needs of the enterprise’s activities and value creation [65, 66]. Many studies have shown that the ability of enterprises to manage operating capital has a profound impact on enterprise performance [67–69]. Green innovation, as an innovative activity, is embedded in the production process of enterprises and is committed to the disruptive transformation or redesign of the original factors of production, and the impact of any small change on the existing system cannot be underestimated [70]. Although in the short term, green innovation has a “double externality” [71], with obvious characteristics of high investment, high risk and low return; however, with the optimisation of production and business processes in the long term, green innovation can significantly reduce compliance costs [72], operating costs [73] and generate additional revenue for the enterprise [74], increasing the accumulation of capital resource base. From the perspective of process innovation, Lu and Li(2023) [50] illustrate the positive role of enterprise green innovation in eliminating outdated and energy-consuming processes, which can use fewer factor resources and time cost inputs to produce the same quality and quantity of products, reduce production costs [18, 51]. Porter et al. (1995) [75] argue from a product innovation perspective that the use of waste and renewable resources in green innovation has a positive impact on cost reduction. Simultaneously, green product innovation through continuous development of new market areas can increase a company’s market share and product sales revenue [76, 77], thereby further increasing the disposable strength of the enterprise’s operating capital. Furthermore, the implementation of green innovation within the patent field has been influential in providing enterprises with sustainable competitive benefits and encouraging their growth [51] through the acquisition of patent transfer advantages [78, 79]. From an innovation economics perspective, green innovation can lower production and operational costs, boost production and market share, and yield earnings from technology licensing [78–80]. This is a reflection of the optimisation of the efficiency of factor allocation in the enterprise, which is conducive to further economic efficiency [51]. Operating capital management is related to every daily aspect of a enterprise, such as supply, production, sales, enhancing competitiveness, and transformational development [81]. The amount of operating capital is the foundation of operating capital management. Filbeck and Krueger’s (2005) study [82] suggests that determining the optimal balance for funds closely related to operating capital is a primary goal of operating capital management, which is crucial for the survival and development of an enterprise and has an important impact on operating capital management decisions. Green innovation not only leads to an increase in resources, but also offers a distinct advantage in optimizing resource allocation and promoting sustainable usage. Digital transformation is challenging and gradual, demanding a high level of resource investment in terms of initial access to support and the efficient allocation and sustainable utilization of resources [11]. Therefore, it can be deduced from the above analysis that green innovation helps enterprises accumulate sales revenue, patent transfers, and a financial foundation resulting from cost reductions,thereby enhancing the enterprise’s ability to manage its operating capital, achieve a reasonable and effective reallocation of resources, subsequently increase the likelihood that the enterprise undertake high-input activities such as digital transformation. This leads to hypothesis 3.

Hypothesis 3: Under the same other conditions, enterprises actively carry out green innovation, which is conducive to the accumulation of capital resources by optimizing production operations, thus affecting the management of operating capital and further influencing the digital transformation of enterprises.

## 3 Data and models

### 3.1 Sample selection and data sources

This paper examines all A-share listed companies from 2010 to 2020 as the research subject. The data derives primarily from the Cathay Pacific database, the CNRDS database, and the Wind database, with some secondary processing of selected variables to suit the research needs. To improve sample comparability and build upon pre-existing research, this article excludes ST, PT enterprises, financial industries, and missing-value samples. Continuous variables are adjusted using a 1% tail shrinking approach to eliminate extreme value effects. The resulting sample includes 20,734 valid observations. Additionally, the study clusters the sample at the firm level to enhance analysis accuracy.

### 3.2 Variable definition

#### 3.2.1 Dependent variable

Enterprise digital transformation (DT). According to Qi et al. (2020) and Zhang et al. (2021) [83, 84], the notes to the financial reports of listed companies disclose the proportion of the digital transformation-related portion of the year-end intangible asset breakdown items to the total intangible assets. This serves as a measure of the digitalisation level of enterprises. Specifically, when the detailed item of intangible assets includes keywords related to Digital transformation technology such as “software”, “network”, “client”, “management system”, “intelligent platform”, as well as related patents, the detailed item is defined as “digital technology intangible assets”, and then several digital technology intangible assets of the same company in the same year are summed up to calculate their proportion in the intangible assets of the current year, The final result is determined as the proxy variable of the degree of digital transformation of the enterprise and expressed by DT. Effective use of digital technology was additionally chosen as a proxy measure for evaluating the degree of digital transformation within the enterprise, for the sake of conducting robustness testing.

#### 3.2.2 Independent variable

Green innovation (GI). As stated previously, implementing green innovation is capable of enhancing production and business procedures and endorsing establishments by signaling their eco-friendliness, ultimately leading to a constructive outcome of both cost reduction and efficiency elevation. Such a dual effect of cost-cutting and productivity enhancement can be realised. Therefore, it is imperative to guarantee the provision of a sufficient amount of green innovations, as well as to concentrate on enhancing innovative quality to accomplish sustainable development. With this in mind, green innovation quantity and quality are considered as proxy variables for green innovation. The volume of green patent applications is a more accurate representation of the green innovation achievement of enterprises [85, 86]. Specifically, the quantity of green innovation is evaluated by summing up the number of green invention patents and utility model patents [86–88], while the quality of green innovation is measured by considering the number of green invention patent applications exhibiting superior technological content [86]. These metrics are identified as GIN and GIQ, correspondingly.

#### 3.2.3 Mechanism variable

Mechanism variable (MV). This paper selects two indicators, financing constraints and operating capacity, to represent the financial status and management capability of enterprises, respectively. The mechanism variable, referred to as MV, is used to represent these indicators.

Mechanism variable 1: Financing constraints (FC). Usually, financing constraints can be measured using multiple indicators. However, Kaplan and Zingales (1997) [55] were the first to combine operating cash flow, dividend payout ratio, gearing ratio, cash holdings, and Tobin’s Q to construct a comprehensive financing constraint indicator, referred to as the KZ index. This index has been widely utilised. Drawing on Kaplan and Zingales (1997) and Wei et al. (2021) [55, 89], the specific approach is: Each year of the full sample is classified according to operating net cash flow/previous period’s total assets (CF), cash dividends/previous period’s total assets (Div), cash holdings/previous period’s total assets (C), gearing ratio (Lev) and Tobin’s Q (TobinQ). Kz1 is assigned a value of 1 if CF is below the median and 0 otherwise. Kz2 is assigned a value of 1 if Div falls below the median and 0 otherwise. Similarly, kz3 is assigned a value of 1 if C is below the median and 0 otherwise. Kz4 is assigned a value of 1 if Lev is above the median and 0 otherwise, and kz5 is assigned a value of 1 if Q is above the median and 0 otherwise. Calculate the KZ index by adding kz1 to kz2 to kz3 to kz4 to kz5. Employ Ordered Logistic Regression (OLR) to regress the KZ index on CF, Div, C, Lev, and Q, used as independent variables. Estimate the regression coefficients of each variable. Using the above regression model’s estimation results, we can calculate the KZ index of each listed company’s degree of financing constraints. A higher KZ index indicates a higher degree of financing constraints and lower efficiency of financing.

Mechanism variable 2: Operating capacity (OC). Operating capacity can accurately indicate an organisation’s ability to create value through managing operating capital and allocating resources. Currently, commonly used indicators for measuring an enterprise’s operating capacity include the total asset turnover ratio, the current asset turnover ratio, and other individual metrics. Additionally, certain research methods are used to calculate comprehensive indicators for the enterprise asset turnover ratio. It is essential to use these indicators to measure an enterprise’s operating capacity. Comprehensive indicators rely more on human factors compared to a single indicator, which can be heavily influenced by subjectivity. Therefore, this paper utilizes the total asset turnover ratio (the ratio of operating income to average total assets) as a proxy variable for measuring operating capacity, following Cheng et al. (2021) [66].

#### 3.2.4 Control variable

In order to enhance the precision of the investigation, this paper also incorporates a range of control variables into the model. These consist of firm size (Size, the natural logarithm of total assets at the end of the year), leverage, commonly referred to as the gearing ratio (Lev, total liabilities at the end of the year/total assets at the end of the year), return on total assets (ROA), and growth (Growth, current year’s operating income/previous year’s operating income-1). No changes needed as the text adheres to the principles of objective and concise academic writing. Shareholder ownership (Top1, percentage of total shares held by largest shareholder), Tobin’s Q (TobinQ), firm age (FirmAge, the current year minus the year of the company’s establishment plus one, transformed to natural logarithm), and capital intensity (CAPINT, total assets divided by operating income).

### 3.3 Econometrics models

In order to test Hypotheses 1, 2, and 3, and with reference to Baron and Kenny (1986) [90], Hayes (2009) [91], and Wen and Ye (2014) [92] on the mesomeric effect test method, we constructed models (1), (2), and (3) to verify the hypotheses.

$$\begin{array}{c} DT_{i,t}=\alpha_{0}+\alpha_{1}GI_{i,t}+\sum \alpha_{k}Controls_{i,t}+\sum Year+\sum Industry+\epsilon_{i,t} \end{array}$$ (1)

$$\begin{array}{c} MV_{i,t}=\beta_{0}+\beta_{1}GI_{i,t}+\sum \beta_{k}Controls_{i,t}+\sum Year+\sum Industry+\epsilon_{i,t} \end{array}$$ (2)

$$\begin{array}{c} DT_{i,t}=\theta_{0}+\theta_{1}MV_{i,t}+\theta_{2}GI_{i,t}+\sum \theta_{k}Controls_{i,t}+\sum Year+\sum Industry+\epsilon_{i,t} \end{array}$$ (3)

**Fig 1:**
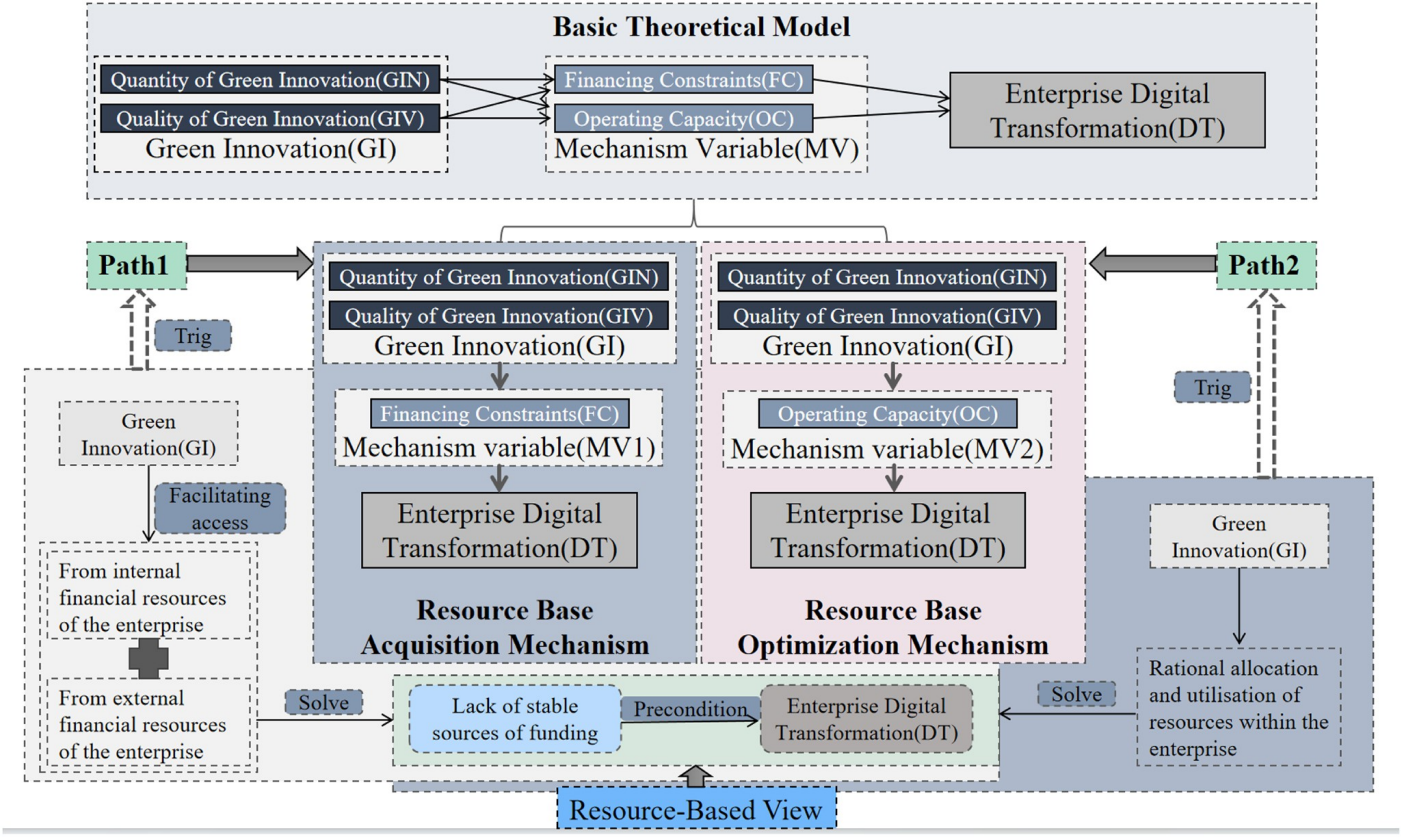
Theoretical model and mechanism path diagram. And the chart is sourced from the author’s own drawing.

In models (1) to (3), DT_i,t_ denotes the interpreted variable, digital transformation. GI_i,t_ represents the explanatory variable for green innovation. MV_i,t_ represents the mediator variable, while Controls_i,t_ is the control variable. The year and industry effects are respectively included in the sum of Year and Industry. Random error term is represented by epsilon. Due to the utilization of two proxy indicators for Green Innovation Quantity (GIN) and Green Innovation Quality (GIQ) in assessing Green Innovation (GI), and two indicators for Financing Constraint (FC) and Operational Capability (OC) as mechanism variables (MV), separate calculations are required in Models (1), (2) and (3) as depicted in Fig 1 to establish the mediation mechanism.

## 4 Empirical analysis

### 4.1 Descriptive analysis

Prior to conducting regression analysis, it is common practice to carry out a descriptive analysis of the sample. Such an analysis allows for the examination of statistical indicators, providing insight into the nature and distribution characteristics of the sample. This process serves as a crucial foundation for the smooth progression of subsequent research. Based on the necessary data for this study, descriptive statistical analysis was performed on the research sample. The results are presented in Table 1. It is evident from the table that the mean values of GIN, GIQ, and DT are relatively low, indicating that there is considerable scope for enhancement of enterprises’ green innovation and digital transformation. With the digital economy becoming the mainstay of future economic development, it is a common trend for enterprises, as leaders of the market economy, to actively undertake digital transformation. Nonetheless, historical experience shows that overemphasizing economic development can easily disrupt the environmental and economic equilibrium. Therefore, finding new development strategies that balance the environment and the economy will continue to pose a challenge for enterprises in their future decisions.

**Table 1:** Descriptive statistical results.

Variables	Obs	Mean	Std. Dev.	Min	Max
*DT*	20,734	0.0932	0.219	0	1
*GIN*	20,734	2.280	7.170	0	52
*GIQ*	20,734	1.268	4.438	0	34
*FC*	20,734	0.724	2.020	-5.417	5.110
*OC*	20,734	0.673	0.438	0.0811	2.490
*Size*	20,734	22.13	1.285	19.95	26.10
*Lev*	20,734	0.416	0.207	0.0536	0.871
*ROA*	20,734	0.0484	0.0539	-0.144	0.206
*Growth*	20,734	0.187	0.363	-0.499	2.136
*Dual*	20,734	0.272	0.445	0	1
*Top*1	20,734	0.355	0.149	0.089	0.745
*TobinQ*	20,734	1.986	1.136	0.904	7.233
*FirmAge*	20,734	2.803	0.352	1.609	3.434
*CAPINT*	20,734	2.373	1.771	0.401	11.128

Note: All table data in this paper are drawn by the author himself. The following text is the same.

### 4.2 Benchmark regression

Table 2 presents the findings of the primary examination of environmentally friendly innovation and its impact on enterprises’ digital evolution. In the initial regression, enterprises’ digital transformation (DT) is employed as the independent variable. Green innovation quantity (GIN) and green innovation quality (GIQ) are used as substitutes for the dependent variable green innovation (GI) in model (1) of the regression. Additionally, the regression includes controls for year and industry effects and a set of control variables. The regression coefficients have all passed the test for statistical significance at the 1% level. The findings demonstrate that both the augmentation in the amount and standard of green creativity substantially improves the digital transformation performance of enterprises. As stated previously, green innovation possesses clear “green” features. It is highly valuable for streamlining production and business procedures, aids in lowering expenses while boosting effectiveness and promotes the formation and use of lasting worth. Moreover, it allows for the accumulation and value addition of an enterprise’s resource base, thus supporting digital advancement. Therefore, Hypothesis 1 has been confirmed. Although the value added per unit of green innovation quantity and quality has a minimal impact on promoting digital transformation, the law of “increasing contribution” holds significant economic importance as unit quantity increases. Although the value added per unit of green innovation quantity and quality has a minimal impact on promoting digital transformation, the law of “incremental contribution” holds significant economic importance as unit quantity increases. It should be noted that when using technical terms, abbreviations should be explained as they are introduced.

**Table 2:** Green innovation and enterprise digital transformation.

	DT	
	(1)	(2)
*GIN*	0.001*** (3.09)	
*GIQ*		0.001*** (3.34)
*Constant*	0.414*** (12.26)	0.419*** (12.30)
*Controlvariables*	Yes	Yes
*Year*/*Industryeffect*	Yes	Yes
*Obs*	20,734	20,734
*adj*.*R*^2^	0.248	0.248

Note: (1) ***, **, * respectively represent significant levels at 1%, 5%, and 10%; (2) The content in parentheses represents the t-value adjusted by clustering robust standard error. The following text is the same.

### 4.3 Robust test

#### 4.3.1 Robustness tests for endogeneity problems

To reduce the influence of endogeneity on the results, this study extends the time frame for examining the effects of green innovation on enterprises’ digital transformation, as shown in Table 3. As an approach, model (1) utilises lagged core explanatory variables (GIN and GIQ) by one period. The findings presented in Table 3 demonstrate that green innovation continues to positively impact enterprises’ digital transformation over the extended observation period. The regression coefficients are positive and successfully pass the 10% and 5% statistical significance tests.

**Table 3:** Robustness test results for extended observation windows.

	DT	
	(1)	(2)
*GIN*	0.000* (1.71)	
*GIQ*		0.001** (2.07)
*Constant*	0.327*** (9.10)	0.332*** (9.16)
*Controlvariables*	Yes	Yes
*Year*/*Industryeffect*	Yes	Yes
*Obs*	17,279	17,279
*adj*.*R*^2^	0.252	0.253

#### 4.3.2 Two-way causal robustness test

Previous theoretical analyses and benchmark regression results have demonstrated that increasing the level of green innovation will drive the progress of enterprise digital transformation and boost its performance. Nevertheless, the investigations by Li et al. (2022) and Luo et al. (2023) [93, 94] indicated that digital transformation also notably improves green innovation performance. To tackle the issue of two-way causality in the study, we adopt the industry averages of the independent variables GIN and GIQ as the instrumental variables for the amount (GIN) and quality (GIQ) of green innovation, respectively, following the standard practice of most scholars in this area. On the one hand, the green innovation level of the industry in which an enterprise is situated may influence its green innovation behavior, ultimately impacting the enterprise’s green innovation performance and fulfilling the instrumental variable correlation hypothesis. On the contrary, the industry’s average level of green innovation principally impacts companies’ choices and behaviours concerning green innovation but does not directly influence their digital transformation process. This finding fulfils the exogeneity hypothesis of the instrumental variable. Table 4 outlines the findings of the second stage of instrumental variable regression analyses. In both models (1) and (2), the Kleibergen-Paap rk LM successfully passes the significance test at the 1% statistical level, thereby rejecting the initial hypothesis of inadequate instrumental variable identification. Similarly, both the Cragg-Donald Wald F-statistics exceed the critical value of 16.38 for the Stock-Yogo F-test for weak instrumental variables at the 10% significance level, which nullifies the original assumption of weak instrumental variable. Therefore, it indicates that the instrumental variables chosen in this paper are justified. Moreover, the regression coefficients of GIN and GIQ are both significantly positive, which reaffirms the fundamental conclusion that environmentally-friendly innovation enhances enterprise digital transformation performance.

**Table 4:** Robustness test results for two-way causation problem.

	(1)	(2)
	DT	DT
*GIN*	0.009* (1.67)	
*GIQ*		0.018* (2.18)
*Kleibergen*−*PaaprkLMstatistic*	96.887***	106.642***
*Cragg*−*DonaldWaldFstatistic*	239.988 [16.38]	306.768 [16.38]

#### 4.3.3 Robustness test on sample selection bias

To minimize the impact of sample selection bias on causal inference results, this paper employs propensity score matching (PSM) for robustness testing [95]. To ensure the reliability of the PSM results, this paper defines samples with green innovation quantity (GIN), and green innovation quality (GIQ) above the median as the experimental group, and samples below the median as the control group, respectively. After the sample grouping, the PSM radius matching method was used for processing, and a matching quality test was conducted on the overall sample. Finally, the causal relationship between the explanatory variables (GIN and GIQ) and the dependent variable (DT) was inferred for the matched samples, as shown in Table 5. The coefficients of GIN and GIQ in columns (1) and (2) of Table 5 are significantly positive at the 1% and 5% statistical levels, indicating that the sample selection issue did not lead to significant estimation errors, and the baseline regression results remain reliable.

**Table 5:** Robustness test results using the PSM method.

	DT	
	(1)	(2)
*GIN*	0.001*** (3.10)	
*GIQ*		0.001*** (3.33)
*Constant*	0.413*** (12.09)	0.419*** (12.28)
*Controlvariables*	Yes	Yes
*Year*/*Industryeffect*	Yes	Yes
*Obs*	20,558	20,702
*adj*.*R*^2^	0.246	0.247

#### 4.3.4 Other robustness tests

A. Methods for replacing variables. In order to further validate the precision of the model, we conduct robustness tests using the replacement of dependent and independent variables as our selected method. Digital transformation, following Wu et al.’s (2021) [96] methodology, involves searching, matching, and counting the frequency of feature words related to artificial intelligence, blockchain, cloud computing, and big data technologies in the annual report. This process classifies and summarizes the word frequencies of the key technological directions, ultimately yielding a metric (DT1) that reflects the effective implementation of digital technology and serves as an alternative measure of the enterprise’s digital transformation. As for the quantity of green innovation and the quality of green innovation, the replacement variables lnGIN and lnGIQ were obtained by following the approach of most studies to add 1 to the variables to take the logarithm for the model robustness test. Columns 1 and 2 of Table 6 show the regression results with the replacement of the dependent variables. The regression coefficients of GIN and GIQ on DT1 are each significantly positive at the 1% statistical level. Columns 3 and 4 of Table 6 show the regression results with the independent variables replaced. lnGIN and lnGIQ regression coefficients of 0.004 and 0.005 on DT pass the significance test at the 5% and 1% levels respectively. The results in Table 6 show that the accuracy of the model is validated in a statistically significant way whether the dependent or independent variables are replaced.

**Table 6:** Robustness test results of substituted dependent variable and independent variable.

	Replace the dependent variable		Replace the independent variable	
	DT1	DT1	DT	DT
	(1)	(2)	(3)	(4)
*GIN*	0.679*** (6.75)			
*GIQ*		1.394*** (7.96)		
*lnGIN*			0.004** (2.49)	
*lnGIQ*				0.005*** (2.81)
*Constant*	-15.608 (-1.20)	-6.393 (-0.49)	0.412*** (12.34)	0.418*** (12.35)
*Controlvariables*	Yes	Yes	Yes	Yes
*Year*/*Industryeffect*	Yes	Yes	Yes	Yes
*Obs*	20,734	20,734	20,734	20,734
*adj*.*R*^2^	0.389	0.390	0.248	0.248

B. Methods for adding missing variables. Restricted by objective conditions such as data availability, it is often straightforward to overlook many additional factors influencing the dependent variable in empirical studies. Thus, to minimise bias caused by omitted variables, this study introduces enterprise market competitiveness and degree of marketization as control variables in the main effects regression. Columns 1 and 2 of Table 7 show the regression outcomes integrating the missing variables. The regression coefficients of GIN and GIQ on DT are positively significant at the 5% and 1% levels, respectively. Therefore, the conclusion that green innovation improves digital transformation’s effectiveness aligns with prior research.

**Table 7:** Robustness test results by adding missing variables and changing sample size.

	(1)	(2)	(3)	(4)
	DT	DT	DT	DT
*GIN*	0.000** (2.43)		0.001*** (2.60)	
*GIQ*		0.001*** (2.73)		0.001*** (3.03)
*Constant*	0.368*** (10.70)	0.373*** (10.75)	0.524*** (10.41)	0.534*** (10.49)
*Controlvariables*	Yes	Yes	Yes	Yes
*Year*/*Industryeffect*	Yes	Yes	Yes	Yes
*Obs*	20,734	20,734	9,578	20,734
*adj*.*R*^2^	0.249	0.249	0.260	0.249

C. Approaches to changing sample size. Although the concept of digital transformation has long been established, it was not until 2016 that the digital economy began to gain national strategic significance. China rapidly advanced the construction of information technology in various areas, continuously consolidating its digital foundations and promoting the concept of digitisation. The government, led by policies, emphasised the importance of digital technology and began to accelerate the development and application of new digital technologies, as well as promoting their integration with the real economy. Since then, enterprises have embraced the current trend and joined the movement towards digital transformation, causing it to flourish and develop. As a result, the research sample period has been revised to cover 2016–2020, and the model is being retested for its robustness. The results align with the baseline regression findings as the regression coefficients of GIN and GIQ on DT in columns 3 and 4 of Table 7 pass the significance test at the 1% statistical level.

## 5 Further discussion

### 5.1 Mechanism verification

Most of the preceding section in this paper has presented ample evidence supporting the discourse on the general influence of green innovation on the digital transformation of enterprises, yet has not substantiated its particular mechanism. According to the theoretical analysis above, green innovation optimises production, emits “green” signals to the capital market, alleviates corporate financing constraints, improves corporate operating capacity, triggers the accumulation of resource base and optimisation mechanisms, and thus promotes the digital transformation of enterprises. To achieve this, regression analyses are conducted using model (1), model (2), and model (3), with green innovation quantity (GIN) and green innovation quality (GIQ) serving as proxies for the independent variable green innovation (GI); financing constraints (FC) and operating capacity (OC) serving as proxies for the mechanism variable (MV); and digital transformation of enterprises serving as the dependent variable. Tables 8 and 9 display the obtained results.

**Table 8:** Test on the intermediate mechanism of green innovation affecting enterprise digital transformation: Financing constraints.

	GIN to FC to DT			GIQ to FC to DT		
	(1)	(2)	(3)	(4)	(5)	(6)
	DT	FC	DT	DT	FC	DT
*GIN*	0.001*** (3.09)	-0.005*** (-3.70)	0.000*** (2.65)			
*GIQ*				0.001*** (3.48)	-0.007*** (-3.35)	0.001*** (2.92)
*FC*			-0.004*** (-3.47)			-0.004*** (-3.47)
*Constant*	0.450*** (12.19)	1.461*** (6.13)	0.422*** (12.47)	0.454*** (12.16)	1.468*** (6.12)	0.427*** (12.50)
*Controlvariables*	Yes	Yes	Yes	Yes	Yes	Yes
*Year*/*Industryeffect*	Yes	Yes	Yes	Yes	Yes	Yes
*Obs*	20,734	20,734	20,734	20,734	20,734	20,734
*adj*.*R*^2^	0.248	0.584	0.248	0.248	0.584	0.248

**Table 9:** Test of the intermediate mechanism of green innovation affecting enterprise digital transformation: Operating capacity.

	GIN to OC to DT			GIQ to OC to DT		
	(1)	(2)	(3)	(4)	(5)	(6)
	DT	OC	DT	DT	OC	DT
*GIN*	0.001*** (3.09)	0.003*** (7.37)	0.000*** (2.66)			
*GIQ*				0.001*** (3.48)	0.005*** (7.79)	0.001*** (2.91)
*OC*			0.007* (1.72)			0.006* (1.68)
*Constant*	0.450*** (12.19)	0.786*** (12.44)	0.411*** (12.23)	0.454*** (12.16)	0.800*** (12.58)	0.416*** (12.27)
*Controlvariables*	Yes	Yes	Yes	Yes	Yes	Yes
*Year*/*Industryeffect*	Yes	Yes	Yes	Yes	Yes	Yes
*Obs*	20,734	20,734	20,734	20,734	20,734	20,734
*adj*.*R*^2^	0.248	0.318	0.248	0.248	0.319	0.248

Tables 8 and 9 confirm the two mechanisms of financing constraints and operational capacity, respectively. The regression coefficients of the independent variables on the dependent variables in columns 2 and 3 and columns 5 and 6 of Table 8 are all significant and negative at the 1% level, indicating that green innovation successfully alleviates enterprises’ financing constraints and consequently improves their digital transformation performance. This also implies that the environmental performance of enterprises significantly contributes to the degree of digital transformation achieved, given the swift progression of the digital economy. The process of digital transformation is time-consuming and demanding, typically entailing substantial investment. An enterprise proficient in environmentally-friendly innovation is more likely to garner market approval, reducing the barrier for support and addressing the deficiency in resources for digital transformation. Therefore, Hypothesis 2 is being tested. The regression coefficients of the independent variables on the explanatory variables in columns 2 and 3, and columns 5 and 6 of Table 9 are significant at the 1% level, and the path of “green innovation to operational capability to digital transformation” is identified as positive. Hypothesis 3 is verified. Green innovation is a potent means for internally optimizing production, reducing costs, and boosting efficiency. As a result, production resources are conserved, resources are allocated and utilised more efficiently and digital transformation performance is improved.

This paper constructed an intermediary effect model using the stepwise regression method, confirming that green innovation helps enterprises alleviate financing constraints, enhance operating capacity, and promote digital transformation. To further verify the intermediary effect of financing constraints (FC) and operating capacity (OC), this paper used Sobel test and Bootstrap test to verify the indirect effects. The results in Table 10 show that the Z statistics of both the Sobel test and Bootstrap test pass significance tests at the 1% and 5% levels, and the 95% confidence interval of the Bootstrap test does not include 0. Both test results indicate that financing constraints (FC) and operating capacity (OC) play an intermediary role in the relationship between enterprise green innovation and digital transformation.

**Table 10:** Results of the Sobel test and Bootstrap test for intermediary effects.

		Sobel test			Bootstrap test			
		(1)	(2)	(3)	(4)	(5)	(6)	(7)
Dependent variable	Mechanism variable	Coef.	Std.Err.	Sobel Z	Coef.	Std.Err.	Bootstrap Z	[95% Conf. Interval]
*GIN*	FC	-0.00006	0.00003	-1.949*	-0.00006	0.00003	-2.03**	[-0.0001,-0.000001]
	OC	0.00020	0.00011	1.769*	0.00025	0.00012	2.13*	[0.00002, 0.0005]
*GIQ*	FC	-0.00006	0.00003	-2.106**	-0.00006	0.00003	-2.18**	[-0.0001, -0.000006]
	OC	0.00026	0.00013	2.039**	0.00026	0.00013	2.01**	[0.000007,0.0005]

### 5.2 Heterogeneity analysis

#### 5.2.1 Heterogeneity of enterprise scale

The paper examines the impact of “green innovation to digital transformation” from a full-sample perspective. However, considering the heterogeneity among firms, the effects of green innovation on digital transformation may show asymmetry in the presence of different firm attributes. Therefore, this study further investigates the outcomes on a split-sample basis. Referring to previous studies, large enterprises refer to those with a higher enterprise size than the mean within the same year. SMEs, on the other hand, refer to those whose enterprise size is equal to or below the mean. Table 11 demonstrates that amongst the large enterprise group, the facilitating effect of green innovation on the digital transformation of enterprises passed the significance test at a 1% level. This paper contends that in comparison to SMEs, large enterprises possess greater scale and resource advantages and sufficient market power. Consequently, these enterprises tend to prioritise the optimisation and upgrading of production and business processes, and have the ability to withstand the high investment and risk of “greening” such processes. They also possess the capacity to govern and allocate their own resources in a rational manner, allowing them to excel in the conversion of environmental performance into economic performance. SMEs, conversely, contend with the fundamental issue of survival and lack the ability to withstand the ramifications of ventures with high-input and high-risk and low-yield. They also possess the capacity to govern and allocate their own resources in a rational manner, allowing them to excel in the conversion of environmental performance into economic performance. As a consequence, these enterprises are unable to effectively convert environmental performance into economic performance through green innovation.

**Table 11:** Heterogeneity of enterprise scale.

	Large enterprises	Minor enterprises	Large enterprises	Minor enterprises
	(1)	(2)	(3)	(4)
	(DT)	(DT)	(DT)	(DT)
*GIN*	0.000*** (3.00)	0.000 (0.40)		
*GIQ*			0.001*** (2.89)	0.001 (1.18)
*Constant*	0.456*** (8.94)	0.674*** (7.42)	0.460*** (8.84)	0.681*** (7.50)
*Controlvariables*	Yes	Yes	Yes	Yes
*Year*/*Industryeffect*	Yes	Yes	Yes	Yes
*Obs*	9,168	11,566	9,168	11,566
*adj*.*R*^2^	0.233	0.267	0.233	0.267

#### 5.2.2 Leverage ratio heterogeneity

Previous research has shown that the level of leverage has a significant effect on innovation performance. This study classifies firms with leverage ratios above the mean as high leverage enterprises and those below as low leverage enterprises. Table 12 displays the outcomes of the grouping by leverage heterogeneity. It has been discovered that green innovation substantially boosts firms’ performance in digital transformation, regardless of their leverage status (all regression coefficients pass the 1% statistical level test and are positively significant). However, the impact of green innovation quantity on enterprises’ digital transformation is more prominent in the high leverage group (regression coefficients are significant at the 5% level). For one thing, the quality of green innovation has made a significant progress from “quantitative change” to “qualitative change”, which conveys “green” signals more clearly and optimises production and business processes. This qualitative shift is more crucial for enhancing the performance of digital transformation than the quantity of green innovations. Hence, it proves to be more effective in improving the digital transformation performance of enterprises. Secondly, a low leverage ratio implies that companies have inadequate solvency, which could easily lead to insufficient financial backing for these companies, thereby impeding their green innovation performance.

**Table 12:** Leverage ratio heterogeneity.

	Low leverage ratio group	High leverage ratio group	Low leverage ratio group	High leverage ratio group
	(1)	(2)	(3)	(4)
	DT	(DT)	(DT)	DT
*GIN*	0.001 (1.53)	0.000** (2.33)		
*GIQ*			0.001** (1.97)	0.001** (2.25)
*Controlvariables*	Yes	Yes	Yes	Yes
*Year*/*Industryeffect*	Yes	Yes	Yes	Yes
*Obs*	10,683	10,051	10,683	10,051
*adj*.*R*^2^	0.281	0.220	0.281	0.220

#### 5.2.3 Regional heterogeneity

Differences in policy, economic, and environmental conditions within a firm’s location result in disparities in their resource base, leading to variations in firm performance. According to Table 13, in the Central region, there is a significant positive correlation between green innovation and digital transformation (Regression coefficients all pass the 1% statistical level test.). Enterprises in the eastern region have a greater overall capacity and abundant resource reserves required for transformation and growth. This makes it easier for businesses to access support from various resources, and creates opportunities for companies to build up a sustained resource reserve. Therefore, in the eastern region the impact of enterprises that provide resource support for the advancement of digital transformation via green innovation to improve digital transformation performance appears to be insignificant. In the western region, the economy suffers from underdevelopment and enterprises lack adequate financial, human and technological resources. This results in low motivation for innovation and challenges in enterprise development, leading to poorer performance. Compared to the eastern and western regions, the central region’s enterprises demonstrate more balanced strengths. They do not suffer the severe innovation limitations of the west and, while somewhat inferior to the eastern region, are able to acquire and create resources. By utilizing green innovation to create and accumulate internal and external resource bases, businesses in the central region can greatly promote their digital transformation. Therefore, the creation and accumulation of internal and external resource bases through environmentally friendly green innovation can greatly facilitate the process of digital transformation for enterprises. The above also highlights the apparent regional imbalances in the co-management of the environment and the economy.

**Table 13:** Regional heterogeneity.

	Eastern region	Central region	Western region	Eastern region	Central region	Western region
	(1)	(2)	(3)	(4)	(5)	(6)
	DT	DT	DT	DT	DT	DT
*GIN*	0.000 (1.04)	0.002*** (4.07)	0.000 (0.30)			
*GIQ*				0.001 (1.39)	0.003*** (4.08)	0.000 (0.91)
*Constant*	0.487*** (11.33)	0.315*** (4.97)	0.075 (0.89)	0.492*** (11.31)	0.314*** (4.98)	0.079 (0.94)
*Controlvariables*	Yes	Yes	Yes	Yes	Yes	Yes
*Year*/*Industryeffect*	Yes	Yes	Yes	Yes	Yes	Yes
*Obs*	20,734	20,734	20,734	20,734	20,734	20,734
*adj*.*R*^2^	0.248	0.318	0.248	0.248	0.319	0.248

## 6 Research conclusions, recommendations and innovations

### 6.1 Research conclusions and recommendations

In recent years, the digital economy has become a more important part of national economic strategy, highlighting the necessity and urgency for enterprise digital transformation. Nevertheless, this transformation is a lengthy and high-risk process that necessitates significant investment and resources. On one hand, digital transformation of enterprises necessitates a stable resource base in the long run; however, it may also lead to a “rough” development style and cause environmental issues. Green innovation, with its dual character of economic and environmental advantages, offers a far-reaching solution to the above problems and promotes the digital transformation of enterprises. Therefore, this paper has chosen a sample of listed companies from 2010 to 2020 to investigate, through empirical methods, the direct impact of green innovation on enterprise digital transformation alongside its intermediate mechanism. The main conclusions obtained are presented below.

Firstly, green innovation significantly enhances enterprises’ digital transformation performance. It should be noted that due to enterprises’ heterogeneity, there is significant variability in the effect of this action. Specifically, when the company scale is larger and the leverage ratio is higher, the promotion effect of green innovation on digital transformation is more prominent. Secondly, companies that engage in green innovations can effectively ease financial constraints, improve operational capacity, and facilitate the accumulation and efficient distribution of resources, which can promote digital transformation within the enterprise. Thirdly, as a result of regional policy, economic and environmental disparities, the coordinated development of enterprise economy and environment exhibits noticeable regional imbalances. Such imbalances are particularly evident between the developed regions in the east and the underdeveloped regions in the west.

Based on the above analysis, this paper suggests three points. Firstly, the government should maintain robust support for businesses, mainly SMEs, to keep up with the evolving times, capitalise on digital transformation prospects and engage in such processes. At the same time, it should encourage businesses to boost production through innovation, actively pursue environmental sustainability, implement “governance while developing” strategies, eliminate environmental issues originating from production sources, and establish a “green + digital economy” mutually beneficial ecosystem to attain high-quality economic growth. Additionally, the government must maintain a balance between controlling leverage and promoting innovation. Studies have found that a greater degree of leverage, on the other hand, enhances innovation performance significantly. Innovation is a key element in national advancement and development, and it remains an endless driving force for enterprise progression and development. Employing innovation to drive production has always been a crucial aspect of the survival process for enterprises. For this reason, there should be no rush to address the issue of enterprise “deleveraging”. Instead, it should be actively and steadily addressed to decrease the leverage ratio of enterprises. Thirdly, the Government should improve its co-ordination efforts to promote the leapfrog development of less developed regions. It should also address the problem of insufficient development between developed regions and less developed regions through better co-ordination. In both the developed east and the less developed west, improving environmental performance has not led to realising effective economic transformation. Thus, each region must identify its own weaknesses, prioritise breakthroughs, address inadequacies, and strengthen the mutual transformation of environmental and economic performance. This is vital to achieving coordinated development of the economy and the environment.

### 6.2 Research innovations

Firstly, this study serves to bridge the gap between the research perspectives of green innovation and digital transformation. After conducting information collection and engaging with the theoretical literature, it was observed that current research primarily focuses on exploring how digital transformation enhances green innovation. However, in the context of the rapid and widespread digital transformation, coupled with the growing societal emphasis on green development, it is crucial to avoid examining the issue from a single perspective alone. In this current landscape, the fundamental objective lies in elucidating the interrelationship between green innovation and digital transformation, and striving to find a pathway for their coexistence. For this purpose, this study adopts an integrated approach that combines theory and empirical methods to investigate the relationship between green innovation and digital transformation. Specifically, it focuses on how green innovation influences digital transformation. This exploration includes verifying the underlying mechanisms, thus bridging the research gap in existing theoretical perspectives on the relationship between green innovation and digital transformation.

Secondly, this study supplements and enriches the relevant content of the resource-based view. The resource-based view plays a crucial role in the long history of enterprise research. Resources are an indispensable foundation for the development of enterprises. This study is based on the resource-based view and further explores the development path of “green innovation obtains resources by alleviating financing constraints to promote digital transformation performance” and “green innovation optimizes resource allocation by enhancing capital operating capabilities to promote digital transformation performance” from the dual perspective of resource-based acquisition and optimization. This is of great significance for the progress and development of resource-based view.

Finally, this study offers valuable insights and serves as a strong reference for achieving coordinated development between the environment and the economy in practice. Therefore, this study begins with the theoretical model of green innovation enhancing digital transformation and further delineates the relationship between environmental benefits and economic gains. Moreover, this study places greater emphasis on the principle of “developing while governing” fully acknowledging the long-term and sustainable of environmental benefits. It aims to explore a high-quality development path for the coordinated governance of the environment and economy in the future.

## Supporting information

S1 Data Data.xlsx is the smallest dataset used to draw conclusions and results from this article. (XLSX)

S1 Text Method.txt is the stata command code that verifies the minimum dataset used to draw conclusions and results from this article. (TXT)
